# Modeling Teacher Supports Toward Self-Directed Language Learning Beyond the Classroom: Technology Acceptance and Technological Self-Efficacy as Mediators

**DOI:** 10.3389/fpsyg.2021.751017

**Published:** 2021-12-16

**Authors:** Xiaoquan Pan, Wei Chen

**Affiliations:** ^1^Xingzhi College, Zhejiang Normal University, Jinhua, China; ^2^College of Foreign Languages, Zhejiang Normal University, Jinhua, China

**Keywords:** self-directed language learning, teacher supports, technology acceptance, technological self-efficacy, English language learning

## Abstract

This study explored the contributions of teacher supports toward students’ self-directed language learning beyond the classroom and investigated whether technology acceptance and technological self-efficacy could be the mediators between teacher supports and students’ self-directed language learning in a sample of Chinese undergraduate students. A total of 197 freshmen students in one university in Eastern China participated in the questionnaires concerning teacher supports, technology acceptance, technological self-efficacy and self-directed language learning. The study highlighted the results: (1) perceived usefulness mediated the relationship between teacher affective supports and students’ self-directed language learning as well as the relationship between teacher capacity supports and students’ self-directed language learning; (2) technological self-efficacy mediated the relationship between teacher affective supports and students’ self-directed language learning as well as the relationship between teacher behavior supports and students’ self-directed language learning; and (3) perceived easy of use had no noticeable mediating functions, but exerted an indirect influence on students’ self-directed language learning. These findings extended previous researches by considering both the external factors (i.e., teacher supports) and the internal factors (i.e., technology acceptance and technological self-efficacy) of influencing students’ self-directed language learning, thereby contributing to enhancing our understanding of the joint drive of the inherent and extrinsic power mechanisms. This study indicated the significance of elevating teachers’ awareness of the substantial supports in enhancing students’ self-directed language learning beyond the classroom and would inform that the future research on teachers’ compliance in relation to technology use be converted from institutional mandates into teachers’ conscientious behaviors.

## Introduction

Technology with its fast-moving pace has pervaded the educational aspects in recent years (Garrison and Akyol, 2009; Hung et al., 2010), thus enabling students’ self-initiated, self-constructed, and self-monitored learning experiences in a newly-constructed technology-based ecology of language learning (Lai and Gu, 2011; Reinders and White, 2011). Online learning, E-learning, M-learning and other informal technological learning approaches provide students with more chances to explore self-directed learning ways (King and He, 2006; Zandi et al., 2014; Hsu, 2016; Huang et al., 2020; Pan, 2020). However, in spite of the booming attention and development on technological teaching approaches in educational landscapes, the enthusiasm and motivation of students to conduct technology-based self-directed language learning need further exploring (Lai et al., 2018). Furthermore, although the technology has become ubiquitous and demonstrated varieties of advantages, how it exerts its strengths and facilitates students’ self-initiated use of technology for language learning is still a sophisticated problem (Chen, 2018; Huang et al., 2019a). Thus, an increasing number of scholars are arguing for the need to provide learners with external support to enhance effective use of technology for language learning (Cohen and White, 2008; Hubbard and Romeo, 2012; Lai et al., 2016). Jeyaraj et al. (2006) found that school factors such as teachers’ influence on technology adoption decisions significantly affected students’ technology-based self-directed learning. According to Huang et al. (2019b), teachers in China are considered as superiors and vital roles in supervising students’ learning, as China is recognized by its collectivist culture where hierarchy is highly appreciated (Hofstede, 2008). Researchers also found that students could increase the frequency of self-initiated use of technology for language learning as a consequence of teachers’ active encouragement and suggestions (Deepwell and Malik, 2008; Lai et al., 2016). Carson and Mynard (2012) further identified that some categories of teacher supports such as pedagogical suggestions, curriculum expectancy contributed to more favorable perception of use of technology and led to greater awareness of language learning potentials. As Lai et al. (2017) pointed out, “given the myriad of ways in which teachers shape language learners’ perceptions of and self-directed use of technology, it is critical to understand how these different types of teacher behaviors interact with other psychosocial factors to influence language learners’ self-directed use of technology for learning outside the classroom” (p. 1107). Research evidence has built up in support of teachers’ supervising behaviors in facilitating students’ willingness to study beyond the classroom (Hagger and Chatzisarantis, 2012). Therefore, teacher supports constitute a multitude of cognitive and non-cognitive functions for stimulating students’ self-directed language learning. In addition to the enhanced external factors that affect students’ technology-based self-directed language learning, various psychological and sociocultural factors that could influence students’ adoption of technological resources for language learning were explored (Bailly, 2011; Lai et al., 2016). For instance, the study of Mew and Honey (2010) indicated that technological learning motivation significantly influences students’ intention to use online learning websites, technology-related facilities and their personal technology application. Among the widely used, multidimensional constructs of perceived behavioral control, technological self-efficacy was considered as the dominant determinant of the intention of using the technology (Teo, 2009; Teo and van Schaik, 2012). However, despite some researches being conducted from either external or internal perspectives, there are still few studies to investigate the influence from both the internal and external factors on students’ self-directed language learning. Therefore, this study aimed to explore how the external factors (i.e., teacher supports) influenced students’ self-directed language learning and whether students’ internal factors (i.e., technology acceptance and technological self-efficacy) would mediate the relationship between teacher supports and students’ self-directed language learning. The present study’s main contribution lies in enhancing our understanding of the potential roles that teachers could play in supporting students’ self-directed use of technology for learning outside the classroom and the joint drive of the inherent and extrinsic power mechanisms.

## Literature Review

### Technology Acceptance Model

Davis (1989) proposed the technology acceptance model (TAM) on the basis of the theory of reasoned action (TRA) raised by Fishbein and Ajzen (1975). “The Technology Acceptance Model (TAM) has been found to be efficient in explaining user behavior across a broad range of end-user computing technologies and user populations” (Teo, 2011, p. 2433). In the TAM, Davis (1989) identified perceived usefulness (PU) and perceived easy of use (PEU) to be the antecedent variables to affect individual’s intentions and behaviors to use technology, as individual’s behavior intention is posted to be affected by the direct and indirect effects of PU and PEU. Perceived usefulness (PU) manifested learners’ expected overall outcome of technology adoption, whereas perceived easy of use (PEU) dominantly pertained to those impacts associated with the process of using technology (Teo, 2011). Perceived usefulness was consistently considered to be the most robust predictor of students’ technology adoption for learning intentions (Yousafzai et al., 2007; Teo, 2011, 2015). Previous research also found that students’ preference and tendency to conduct technology-based learning was determined by their perception of the potential usefulness of technological resources (Clark et al., 2009; Lai and Gu, 2011). Therefore, perceived usefulness was involved as a significant predictor in our hypothesized model. Additionally, in response to Davis’s (1989) conforming perceived ease of use as an antecedent of perceived usefulness, the associations between the two have been further explored. For instance, perceived easy of use (PEU) was examined to have a positive effect on perceived usefulness (PU) (Liaw and Huang, 2003; Teo, 2009; Wong et al., 2012). In the TAM model, “these two constructs influence the user’s Attitude toward using the system (AT), which in its turn influences the Behavioral Intention to use the system (BI), which determines at the endpoint the actual system use where people use the technology” (Papakostas et al., 2021, p. 2). The new integrated TAM model proposed by Venkatesh and Bala (2008) takes into consideration user’s general beliefs (i.e., perceptions of external control, technological self-efficacy) about computer applications. In recent years, the TAM has been widely utilized in many other areas such as economy and pedagogy. In the educational landscape, there are lots of empirical researches to connect pedagogical support for the use of TAM. For instance, Liaw et al. (2007) found out that students’ technology acceptance is the key factors for technology-based learning. Hsieh et al. (2017) also proposed that the technology acceptance was the prerequisite for students to learn knowledge *via* using technology. Besides, there are further studies exploring the associations between learner’s technology acceptance and other factors such as self-efficacy (Cho and Kim, 2013). Lai (2015) investigated the relationship between internal variables of technology acceptance and learner’s intention to use technology in language learning context. Currently, TAM has been identified as a stable and parsimonious theoretical model for applications in educational contexts, such as mobile game-based learning as a solution in COVID-19 era (Krouska et al., 2021), social networking-based learning (Troussas et al., 2021), the integration of Augmented Reality (AR) in course training (Papakostas et al., 2021), the digital learning technologies (Sprenger and Schwaninger, 2021), and language teachers’ adoption of educational technology (Sun and Mei, 2020). However, although there are a lot of studies to explore the technology acceptance model and connect this model with educational issues, there are still few researches to further investigate how learners’ technology acceptance influence language learning in mainland China and whether technology acceptance can be the mediating variable to influence students’ self-directed language learning.

### Teacher Supports

Teachers significantly shape the quality of students’ learning experiences by affecting students’ cognitive, affective and social learning behaviors (Farmer et al., 2011). As a significant social agent, teachers play a critical role in helping students develop autonomy of technology-based language learning beyond class (Reinders and Darasawang, 2012). Knowles (1989) defined self-directed learning as “a process in which individuals take the initiative, with or without the help from others, in diagnosing their learning needs, formulating goals, identifying human and material resources, choosing and implementing appropriate learning strategies and evaluating learning outcomes”(p. 18). Extant literature has indeed approached self-directed learning from the perspectives of the personal attribute (e.g., individuals’ propensity, willingness and capacity to conduct learning behaviors; Garrison, 1997), the process (Salleh et al., 2019), and the context (Song and Hill, 2007). In light of these particular research lines, the function of teacher supports should be manifested in helping students to be academically, professionally and psychologically empowered, motivating students’ personal attribute, and facilitating students’ self-initiated use of technological resources to autonomously clutch the reins of self-directed learning process. According to Fagerlund (2012), in-class technological instructions and supports conducted by teachers will be learned and continued by students outside the classroom. Based on students’ exposure to engaging learning experience and environments, Lai and Gu (2011) found that it was more possible for students to use the technologies that teacher had used in class. Accordingly, both the quantity and quality of students’ autonomous use of technology to learn language are deeply influenced by teachers’ opinions and behaviors (e.g., Arbaugh, 2000; Margaryan and Littlejohn, 2010; Imlawi et al., 2015; Hao et al., 2017). Carson and Mynard (2012) identified different teacher supports that facilitated students self-directed language learning: (1) by raising students’ technological awareness through expounding the advantages of technology in language learning; (2) by offering technological resources/strategies to help students slash the difficulties of discovering useful resources online; and (3) by organizing varieties of technological activities to activate students technological interests. Researches have reported that the guidance and support from teachers drove students’ engagement in technology-based self-directed language learning (Ertmer et al., 2012), helped students incorporate learning resources/activities into their learning ecology (Lai et al., 2014), and facilitated students to utilize technology as learning tools (McLoughlin and Lee, 2010). Due to different characteristics and functions of teacher supports, researchers resorted to the classification of teacher supports so as to definitely depict the associations of teacher supports and students’ learning behaviors. Three categories of teacher supports of technology were posited, respectively, teacher affective supports (Carson and Mynard, 2012; Lai, 2015), teacher behavior supports (Deepwell and Malik, 2008; Gray et al., 2010; Fagerlund, 2012; Lai, 2013) and teacher capacity supports (Fagerlund, 2012; Lai, 2015). Teacher affective supports (TAS) mainly refer to teacher behaviors which can provide students with the basic knowledge of the strengths of technology as well as the encouragement of using technology in language learning (Xia and Lee, 2000). Teacher behavior supports (TBS) involve teachers’ capacities of organizations and management that can help students participate in activities and tasks involving technologies (Ertmer, 2005). Teacher capacity supports (TCS) mainly help students to get some useful technological resources and tell them how to select and use technological resources effectively (Gallivan et al., 2005). The current literature abounds in discussions on the impact of teacher supports on promoting students’ language learning. However, the internal mechanism between teacher supports and students’ technology-based self-directed language learning beyond the classroom needs to be further explored.

### Technological Self-Efficacy

Bandura’s (1997) notion of self-efficacy highlighted how one individual’s self-regulatory process influence his or her behavior, and thereby self-directed learning manifested the degree to which students are “metacognitively, motivationally, and behaviorally active participants in their own learning process” (Zimmerman, 1986, p. 308). Researchers for decades have been conducting studies in understanding the especially important role that self-efficacy plays in connection with self-directed learning. Research evidence shows that self-efficacy has strong relationship with one’s expectations and interests in learning, including the enhancement of one’s confidence (Zuffianò et al., 2013), the improvement of the degree of one’s efforts on tasks (Abali Ozturk and Sahin, 2015), and the perceived responsibility for learning (Kitsantas and Zimmerman, 2009). Further, research result indicated that students with higher self-efficacy demonstrated a higher volley of inspirations and motivations than lower self-efficacy students, and tended to spend more time on their studies (Bassi et al., 2007). In the study of Zuffianò et al. (2013), self-efficacy has academically been viewed to possess the function to allow students to experience the feeling of worth and confidence which can contribute to students’ better learning performance. In the wake of network technology, the phenomenon of combining the self-efficacy with technology is triggered. Based on the concept of self-efficacy, technological self-efficacy mainly refers to one’s perception of his or her capacities to use technology-connected tools or resources to conduct and finish some tasks (Keengwe, 2007). Among the key motivation constructs associated with students’ technology adoption for self-directed learning, technological self-efficacy is identified as the important factor that affects one’s use of technology (Yesilyurt et al., 2016). In this study, technological self-efficacy is characterized as students’ perception of their capabilities to utilize technology-related tools and sites to conduct learning behaviors so as to achieve intended learning outcome (Bandura, 1997; Keengwe, 2007). Researchers have verified a significant positive influence of technological self-efficacy on technology acceptance and utilization (Celik and Yesilyurt, 2013) and regarded technological self-efficacy as a proxy of individuals’ control beliefs in technology use (Venkatesh and Davis, 1996). Researchers have also found that technological self-efficacy significantly affects students’ behavioral preferences to use technological tools and their perceptions of the usefulness of technology for learning (Keengwe, 2007; Mew and Honey, 2010). More specifically, in learning process, technological self-efficacy constitutes a significant psychological homeostasis that students utilize to help develop their habits of using technology and their perceptions of the usefulness of technology in learning (Keengwe, 2007; Mew and Honey, 2010). Therefore, technological self-efficacy noticeably affects students’ technology-related language learning behaviors. As one of the students’ causal attributions regarding their technology-based self-directed learning, this variable of technological self-efficacy should be considered and examined, especially in the technology-correlated educational context.

While a burgeoning research on self-directed language learning, self-efficacy, and sources of self-efficacy has been conducted (Sundqvist, 2011; Su et al., 2018), there remains a lack of research examining both the external and internal variables which influence students’ awareness and perceptions of technology acceptance and technological self-efficacy in cultivating and enhancing their behaviors of self-directed language learning. Based on the previous researches, teachers as the important social agents have the irreplaceable roles in directing and facilitating students’ self-directed language learning (Davis, 2003). Therefore, this study aims to connect more variables (e.g., teacher support, technology acceptance, technological self-efficacy) to explore their respective influence on students’ self-directed language learning and the potential relationships.

### Problem Statement and Hypothesis

Informed by recent new visions in the study of technology-based self-directed language learning discussed above, two research questions are specified below:

Question 1: How do teacher supports contribute to students’ technology-based self-directed language learning beyond the classroom?

Question 2: Will technology acceptance and technological self-efficacy mediate this relationship?

**FIGURE 1:**
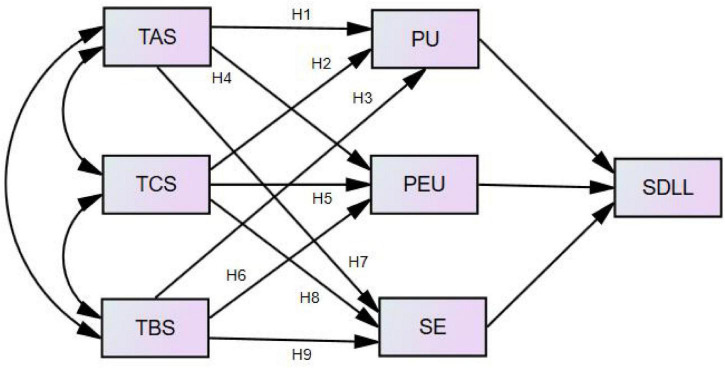
The hypothesis model.

Thereby, this research aimed to test the following nine hypotheses (Figure 1):

H1: Teacher affective support (TAS) correlates with students’ self-directed language learning (SDLL) *via* perceived usefulness (PU).

H2: Teacher capacity support (TCS) correlates with students’ self-directed language learning (SDLL) *via* perceived usefulness (PU).

H3: Teacher behavior support (TBS) correlates with students’ self-directed language learning (SDLL) *via* perceived usefulness (PU).

H4: Teacher affective support (TAS) correlates with students’ self-directed language learning (SDLL) *via* perceived easy of use (PEU).

H5: Teacher capacity support (TCS) correlates with students’ self-directed language learning (SDLL) *via* perceived easy of use (PEU).

H6: Teacher behavior support (TBS) correlates with students’ self-directed language learning (SDLL) *via* perceived easy of use (PEU).

H7: Teacher affective support (TAS) correlates with students’ self-directed language learning (SDLL) *via* technological self-efficacy (SE).

H8: Teacher capacity support (TCS) correlates with students’ self-directed language learning (SDLL) *via* technological self-efficacy (SE).

H9: Teacher behavior support (TBS) correlates with students’ self-directed language learning (SDLL) *via* technological self-efficacy (SE).

## Methodology

### Participants and Procedure

Participants were freshmen students at a large comprehensive university in Eastern China who were taking compulsive college English courses at the time of this study. Currently, the advanced network technology has been applied in college English teaching and learning in accordance with the innovation of college English course. Totally 201 freshmen students voluntarily participated in the survey and were told of their rights to decline. Questionnaires were distributed to the participants on the spot at the class interval of college English course and collected immediately after completion. After consent, participants were briefed several measures of the questionnaire and completed anonymously within 10 min. A total of 197 valid questionnaires were retained after discarding incomplete questionnaires. Of the valid participants, 71 were males (36%) and 126 were females (64%), with an average age of 19 (SD = 1.45). Noticeably, the equipped network technology in this university is accessible to all students, offering them good facilities to independently conduct self-directed language learning beyond the classroom. Hence, the experiences of the participants’ technology adoption for self-directed language learning are representative of what most language learners in this university would experience.

### Measures

The questionnaire items which were adapted and validated from various published sources were used to assess teacher supports (Lai, 2015), technology acceptance (Davis, 1989; Moore and Benbasat, 1991; Taylor and Todd, 1995; Venkatesh and Davis, 2000; Ajzen, 2002), technological self-efficacy (Keengwe, 2007; Celik and Yesilyurt, 2013) and self-directed language learning (Jansen and Janssen, 2017) respectively. Each questionnaire item was measured on a 5-point Likert Scale, ranging from 1 (strongly disagree) to 5 (strongly agree). Higher scores indicated higher perceptions of teacher supports, technology acceptance, technological self-efficacy and self-directed language learning.

### Teacher Supports

Teacher supports were measured in three scales: teacher affective supports (four items, e.g., My English teacher encourages us to use technology for language learning outside the classroom), teacher behavior supports (four items, e.g., My English teacher assigns assignments that are based on or involve the use of online resources or tools), and teacher capacity supports (four items, e.g., My English teacher shares with us useful technological resources/sites/tools for language learning outside the classroom). The Cronbach alpha values of teacher affective supports, teacher behavior supports and teacher capacity supports are 0.916, 0.89, and 0.888, and Kaiser–Meyer–Olkin (KMO) values for validity are 0.846, 0.797, and 0.803, respectively, indicating a good reliability and validity.

### Technology Acceptance

Technology acceptance was measured using two scales: perceived usefulness (five items, e.g., I find technologies useful in language learning) and perceived ease of use (five items, e.g., I find it easy to select and find appropriate technological tools needed to enhance language learning). As the Cronbach alpha values of perceived usefulness and perceived ease of use are 0.915 and 0.857, respectively, and Kaiser–Meyer–Olkin (KMO) values for validity are 0.886 and 0.748, the scale has a good reliability and validity.

### Technological Self-Efficacy

Students’ technological self-efficacy included five items, e.g., I have the confidence to be proficient in using technology when learning English independently. The Cronbach alpha value of technological self-efficacy is 0.909 and Kaiser–Meyer–Olkin (KMO) value for validity is 0.852, indicating that the scale has a good reliability and validity.

### Students’ Self-Directed Language Learning

Students’ self-directed language learning included four items, e.g., I like self-directed English learning outside the classroom. The Cronbach alpha value of students’ self-directed language learning is 0.917 and Kaiser–Meyer–Olkin (KMO) value for validity is 0.853, thereby indicating that the scale has a good reliability and validity.

### Method of Data Analysis

Firstly, SPSS21.0 was used to analyze the reliability and the validity of each variable (i.e., teacher supports, technology acceptance, technological self-efficacy and students’ self-directed language learning). Secondly, data were analyzed using structural equation modeling (SEM) *via* Amos 21.0, including the examining of measurement model and the structural part of the SEM (Teo et al., 2016). Following the recommendations by Hu and Bentler (1999), the model fit was tested by using several goodness-off it indexes, including the ratio of the chi-square to its degrees of freedom (X^2^/df), RMSEA, SRMR, CFI, and TLI. By Hair et al. (2010), values of X^2^/df (<3), CFI (>0.90), TLI (>0.90), RMSEA (<0.08) and SRMR (<0.08) are reflective of a good fit. In addition, the significance of the mediation effects was assessed using the bias-corrected percentile bootstrap method (Hayes, 2013), computing the confidence interval (CI) for the mediated effect. When zero is not in the CI, it indicates the significance of the indirect effect.

## Research Results

### Descriptive Results and Correlations

As is shown in Table 1, the mean values of 7 variables varied from 3.27 to 3.88, indicating participants’ positive response to the variables in the questionnaire. The standard deviations varied from 0.78 to 1.01, which was indicative of a narrow spread of participants’ responses.

**TABLE 1:** Descriptive statistics of study variables.

	N	Minimum	Maximum	Mean	SD
PU	197	1	5	3.79	0.89
PEU	197	1	5	3.88	0.78
TAS	197	1	5	3.76	0.86
TCS	197	1	5	3.80	0.85
TBS	197	1	5	3.45	0.91
SE	197	1	5	3.79	0.90
SDLL	197	1	5	3.27	1.01

*PU, perceived usefulness; PEU, perceived ease of use; TAS, teacher affective supports; TCS, teacher capacity supports; TBS, teacher behavior supports; SE, technological self-efficacy; SDLL, self-directed language learning.*

Tables 2, 3 present that all the measures had good reliabilities (Cronbach’s alpha ranged from 0.857 to 0.917). Pearson correlation matrices for the relations between variables show that there were noticeable correlations among the study variables. As shown in Table 2, TCS and TAS had a relatively high correlation (*r* = 0.826), so collinearity variance inflation factors (VIFs) were calculated to examine potential multicollinearity problems. The VIF scores ranged between 1.832 and 4.361 (all < 5), which indicated that the estimation of the regression coefficients would not be affected by multicollinearity problems (Montgomery et al., 2001).

**TABLE 2:** Correlations among study variables.

Variables	PU	PEU	TAS	TCS	TBS	SE	SDLL
PU	(0.826)						
PEU	0.688**	(0.740)					
TAS	0.579**	0.677**	(0.858)				
TCS	0.561**	0.648**	0.826**	(0.821)			
TBS	0.340**	0.424**	0.647**	0.672**	(0.826)		
SE	0.564**	0.725**	0.558**	0.524**	0.473**	(0.817)	
SDLL	0.518**	0.442**	0.416**	0.473**	0.470**	0.586**	(0.859)

*N = 197. PU, perceived usefulness; PEU, perceived ease of use; TAS, teacher affective supports; TCS, teacher capacity supports; TBS, teacher behavior supports; SE, technological self-efficacy; SDLL, self-directed language learning. Diagonal in parentheses: square root of average variance extracted from observed variables (items).*

**p < 0.01.

**TABLE 3:** The convergent and discriminant validity of the measurement model.

Constructs	Items	Standardized factor loading	CR (>0.7)	AVE (>0.5)	Cronbach’s alpha
PU	PU1	0.795	0.915	0.683	0.915
	PU2	0.871			
	PU3	0.805			
	PU4	0.848			
	PU5	0.816			
PEU	PEU1	0.596	0.857	0.547	0.857
	PEU2	0.701			
	PEU3	0.785			
	PEU4	0.800			
	PEU5	0.830			
TAS	TAS1	0.826	0.918	0.737	0.916
	TAS2	0.832			
	TAS3	0.905			
	TAS4	0.868			
TCS	TCS1	0.820	0.891	0.674	0.888
	TCS2	0.891			
	TCS3	0.825			
	TCS4	0.736			
TBS	TBS1	0.857	0.895	0.682	0.892
	TBS2	0.850			
	TBS3	0.855			
	TBS4	0.734			
SE	SE1	0.819	0.91	0.668	0.909
	SE2	0.825			
	SE3	0.839			
	SE4	0.852			
	SE5	0.757			
SDLL	SDLL1	0.848	0.918	0.738	0.917
	SDLL2	0.896			
	SDLL3	0.876			
	SDLL4	0.811			

### Measurement Model

Confirmatory factor analysis (CFA) was conducted to assess the fitness of this measurement model. Firstly, to assess the discriminant validity, the square root of AVE for each construct was tested. “If the square root of the AVE of a construct was greater than the off-diagonal elements in the corresponding rows and columns, this suggests that a construct is more strongly correlated with its indicators than with the other constructs in the model thus suggesting the presence of discriminant validity” (Teo, 2011, p. 2436). Table 2 demonstrated that this measurement model established the discriminant validity, as the square root of AVE (shown in parentheses along the diagonal) of each construct is higher (0.740–0.859) than corresponding correlation values for that variable in all cases. Secondly, the convergent validity of the measurement model was tested by examining the reliability of each item through its factor loading and assessing the construct reliability by the Cronbach’s alpha, and average variance extracted (AVE), t-value (C. R. > 2) and S. E. value (>0) of parameter estimation. Teo and van Schaik (2012) suggested the standardized factor loadings exceed 0.7, and average variance extracted (AVE) by each construct exceed 0.50. By these criteria, Table 3 indicated good convergent validity of this measurement model. In addition, Table 3 indicated that, except for PEU1, the standardized factor loadings for all the study constructs exceeded the minimum of 0.70, suggesting good construct validity. PEU1 was not excluded from further analysis because it was statistically significant.

### Path Analysis Testing the Hypothesized Model

Grounded on the previous researches (e.g., Hu and Bentler, 1999; Marsh et al., 2004), this study examined the model fit using the root mean square error of approximation (RMSEA), the standardized root mean square residual (SRMR), comparative fit index (CFI), and the Tucker–Lewis index (TLI). A good model is indicated by RMSEA < 0.08, SRMR < 0.06, and CFI and TLI > 0.90.

**FIGURE 2:**
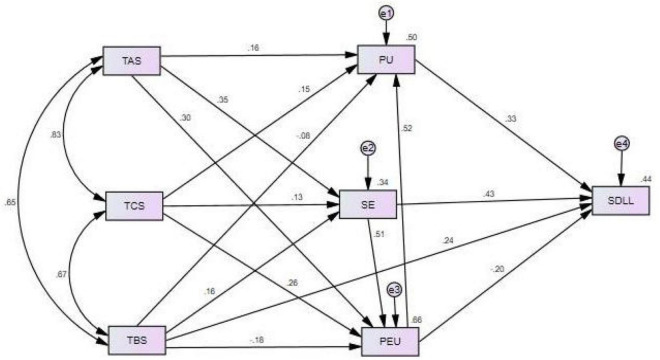
The modified model.

As is shown in Table 4, the unrevised model didn’t significantly satisfy the fitting standard values. According to the modification indices in AMOS 21.0, The M. I. values of the paths of technological self-efficacy (SE)→perceived easy of use (PEU), perceived easy of use (PEU)→technological self-efficacy (SE) and teacher behavior supports (TBS)→self-directed language learning (SDLL) are 43.801, 33.864 and 10.78,indicating that a better model can be established by adding these three paths. Therefore, after adding the three paths, the modified structural model (Figure 2) yielded a better fit (X^2^/df = 2.616 < 3, GFI = 0.908 > 0.90, CFI = 0.991 > 0.90, RMSEA = 0.079 < 0.08, SRMR = 0.0154 < 0.06).

**TABLE 4:** Comparison of fitting test value and fitting standard value of the modified hypothesis model.

	CMIN/DF	GFI	CFI	RMSEA	SRMR
Fitting standard value	<3 is good	>0.9	>0.9	<0.08 is good	<0.06
Unrevised model	25.212	0.816	0.836	0.351	0.1144
Added: SE→PEU	14.126	0.915	0.926	0.259	0.0812
Added: PU→PEU	5.640	0.969	0.979	0.154	0.0427
Added: TBS→SDLL	2.616	0.989	0.995	0.079	0.0154

*CMIN/DF, Chi-square/Degrees of freedom.*

According to Table 5, except teacher capacity supports (TCS)→technological self-efficacy (SE), teacher affective supports (TAS)→perceived usefulness (PU), teacher capacity supports (TCS) → perceived usefulness (PU), teacher behavior supports (TBS) → perceived usefulness (PU), the standardized path coefficient of the other paths is not close to or greater than 1, and the parameter estimation SE value is greater than 0, indicating that the parameters of the structural model are reasonable; the CR critical value is greater than 2, and the *p* value is significant under the level of 0.005, indicating that the parameters of the structural model are significant.

**TABLE 5:** The path analysis.

Path	Path coefficient	S. E.	C. R.	*P* (<0.05)	Results
TCS → SE	0.137	0.116	1.189	0.234	Not support
TAS → SE	0.359	0.110	3.261	0.001	Support
TBS → SE	0.160	0.079	2.020	0.043	Support
TBS → PEU	–0.158	0.050	–3.141	0.002	Support
TAS → PEU	0.271	0.071	3.828	***	Strongly support
TCS → PEU	0.238	0.073	3.281	0.001	support
SE → PEU	0.446	0.045	9.988	***	Strongly support
TAS → PU	0.162	0.100	1.613	0.107	Not support
TCS → PU	0.159	0.102	1.560	0.119	Not support
TBS → PU	–0.082	0.068	–1.197	0.231	Not support
PEU → PU	0.587	0.080	7.381	***	Strongly support
PU → SDLL	0.377	0.084	4.497	***	Strongly support
SE → SDLL	0.486	0.091	5.373	***	Strongly support
PEU → SDLL	–0.255	0.119	–2.141	0.032	Support
TBS → SDLL	0.265	0.068	3.901	***	Strongly support

Path coefficient = standardized path coefficient. ***p < 0.001.

### Assessment of Mediating Paths

Table 6 presented that TAS → PU → SDLL, TAS → SE → SDLL and TCS → PU → SDLL had total mediating effects. In addition, TBS → SE → SDLL had partial mediating effects. As such, it can be found that perceived usefulness (PU) and technological self-efficacy (SE) mediated the relationship between teacher supports and self-directed language learning (SDLL) with a statistically significant 95% confidence interval (CI) values. According to the guidelines by Cohen (1988), effect sizes with values less than 0.1 are considered small, those with less than 0.3 are medium, and values with 0.4 or more are considered large. The results of the mediation analysis shown in Table 6 indicated statistically significance and accorded with the guidelines by Cohen (1988) with small to medium (0.079 < 0.178) indirect effect values.

**TABLE 6:** The mediating paths.

Items	C total effects	a	b	a × b mediating effects	a × b (95% Boot CI)	C’ direct effects	Results
TAS → PU → SDLL	–0.002	0.415**	0.379**	0.157	0.043∼0.232	–0.232	Total mediating effects
TAS → PEU → SDLL	–0.002	0.431**	–0.244	–0.105	−0.195∼0.008	–0.232	No significant mediating effects
TAS → SE → SDLL	–0.002	0.359**	0.496**	0.178	0.014∼0.325	–0.232	Total mediating effects
TCS → PU → SDLL	0.345*	0.335**	0.379**	0.127	0.022 ∼ 0.223	0.223	Total mediating effects
TCS → PEU → SDLL	0.345*	0.299**	–0.244	–0.073	−0.150∼ 0.006	0.223	No significant mediating effects
TCS → SE → SDLL	0.345*	0.137	0.496**	0.068	−0.059∼ 0.165	0.223	No significant mediating effects
TBS → PU → SDLL	0.310**	–0.132	0.379**	–0.05	−0.116∼ 0.005	0.259**	No significant mediating effects
TBS → PEU → SDLL	0.310**	–0.086	–0.244	0.021	−0.014∼ 0.067	0.259**	No significant mediating effects
TBS → SE → SDLL	0.310**	0.160*	0.496**	0.079	−0.003∼ 0.160	0.259**	Partial mediating

C stands for total effects without mediating variables, a stands for regression coefficient of independent variables → mediating variables, b stands for regression coefficient of mediating variables → dependent variables, c’ stands for regression coefficient with mediating variables (i.e., direct mediating effect) of independent variables → dependent variables, *P < 0.05; **P < 0.01.

## Discussion

Currently, technology is increasingly utilized in Chinese classrooms. This increase in technology access lessens external barriers known as first-order barriers (Kopcha, 2012). Previous studies which have primarily focused on teachers’ technological integration into pedagogical instructions found that technology access does not automatically equate to high efficiency of technology usage (Ertmer and Ottenbreit-Leftwich, 2010) and that teachers are still limited in facilitating students’ technology-based learning (Buabeng-Andoh, 2012). Thereby, the role value on teachers’ internalization of external barriers and externalization of personal beliefs for technology integration was highlighted (Vongkulluksn et al., 2018), and more importantly, “it is essential that we not only focus on what teachers could do with technologies inside the classroom but also explore how teachers could help maximize the potentials of technology for learning by enhancing the quantity and quality of learner self-directed use of technology for learning outside the classroom” (Lai, 2015, p. 80).

The present study investigated the mediating roles of internal factors linking teachers’ various supports to students’ self-directed language learning. Specifically, the study constructed a multiple mediation model to examine the mediating roles of perceived usefulness, perceived easy of use and technological self-efficacy in the associations between teacher supports and students’ self-directed language learning. The results demonstrated that three categories of teacher supports influenced the development of mediating factors (i.e., perceived usefulness, perceived easy of use and technological self-efficacy) which subsequently linked to students’ self-directed language learning. These findings extended previous researches by considering both the internal factors (i.e., perceived usefulness, perceived easy of use and technological self-efficacy) and external factors (i.e., teacher supports) of influencing students’ self-directed language learning. Consistent with previous study, the path analysis revealed that teacher supports, especially teacher behavior supports, were directly associated with students’ self-directed language learning (Lai, 2015). Specifically, students who perceive teachers’ behavior supports such as teachers’ encouragement to use technology resources tend to conduct more self-directed language learning beyond the classroom. The path analysis of this study also indicated that the other two teacher supports (i.e., teacher affective supports and teacher capacity supports) didn’t exert the direct influence on but affected students’ self-directed language learning as the mediating factors. Affective support and capacity support such as encouragement, the recommendations of learning resources and the instructions of metacognitive strategies are the responsibilities of teachers that have been found to be largely unaware of Toffoli and Sockett (2015). Additionally, the multiple mediating model indicated how the teacher supports indirectly influenced students’ self-directed language learning through the mediating role of students’ perceived usefulness, perceived easy of use and technological self-efficacy. Thus, an implication of the results for professional development initiatives is that teacher supports need to be highlighted as: (1) undertaking teachers’ responsibilities of facilitating students’ willingness and capacities for technology-based learning in and beyond the classroom; (2) providing scaffolding mechanisms of supporting students’ self-directed use of technology for learning outside the classroom (Reinders, 2010); and (3) incarnating teachers’ capacity of maximizing the potentials of technology for education.

### The Mediating Role of Technology Acceptance

The results of this study revealed that perceived usefulness mediated the relationship between teacher supports and students’ self-directed language learning. According to the mediating paths, perceived usefulness totally mediated the relationship between teacher affective supports and students’ self-directed language learning as well as the relationship between teacher capacity supports and students’ self-directed language learning. The former mediating path coincided with the previous study which considered verbal persuasion or affective support as an important antecedent to induce people’s behavioral changes through their positive attitudinal changes (Petty and Cacioppo, 1986). Lai (2015) identified that teacher affective supports could predict self-directed technology use by improving students’ perceived usefulness. Therefore, teachers’ affective supports such as oral persuasion and encouragement can induce students’ behavioral changes such as changing their self-directed language learning behaviors through students’ positive attitudinal changes (e.g., their perceived usefulness). The latter mediating path revealed that perceived usefulness had the mediating influence on the relationship between teacher capacity supports and students’ self-directed language learning, which is also accordance with the previous study (Lai, 2015). Additionally, the mediating role of perceived usefulness between teacher capacity support and students’ self-directed language learning corroborated previous studies concerning the role value of teacher capacity by: (1) improving students’ awareness of usefulness for the behavior (Xia and Lee, 2000); (2) strengthening students’ willingness to use variety of and potentials of technological resources to learn the language outside the classroom (Gamble et al., 2012); and (3) facilitating students to learn language more positively and independently after class (Lai, 2015). For instance, teachers’ capacity behaviors such as providing in locating, selecting and using appropriate technological resources had indirect influence on their behaviors by changing students’ behavioral intentions (McLoughlin and Lee, 2010; Lai et al., 2016). Students may enhance their perceived usefulness of technology and increase the frequency of self-directed language learning outside the classroom on the condition that teachers offer students capacity supports.

The results of this study found no noticeable mediating functions of perceived easy of use between teacher supports and students’ self-directed language learning and supported the hypotheses by previous researches (e.g., Davis, 1989; Venkatesh et al., 2003; Lai et al., 2012) which confirmed that perceived easy of use didn’t have direct effects on user’s behavioral intention but indirectly influenced user’s intentions to use technology through perceived usefulness. Students’ self-directed language learning can be facilitated by directly enhancing their perceived usefulness and indirectly strengthening their perceived easy of use. In this study, according to the path analysis of teacher affective supports to perceived easy of use, teacher affective supports strongly promoted perceived easy of use, which verified the previous study that teachers’ verbal persuasion or oral encouragement had the positive influence on students’ perceived easy of use because teachers are the critical pedagogical examples and agents that shape students’ awareness to use technology (Reinders, 2010; Toffoli and Sockett, 2015). Thus, this study echoed the view of Teo and Noyes (2014) that “technology providers ensured the ease of use of media which are targeted at teaching and learning in order to attract more educational users” (p. 62).

### The Mediating Role of Technological Self-Efficacy

The study also documented that both teacher affective supports and teacher behavioral supports could relate to students’ self-directed language learning through the mediating role of technological self-efficacy. According to Henry (2013), perceived social support had a significant influence on technological outcome expectations and interests. What’s more, technological self-efficacy had strong associations with technological expectations and interests (Sheu et al., 2010). Fishbein et al. (1980) conceptualized that verbal persuasion or affective support was the fundamental element for individuals’ attitudinal beliefs and confidence. Technological self-efficacy which standards for individuals’ intentions and beliefs to use technology was considered as the vital determinant of the behavior of using the technology (Hall and Higgins, 2005; Ma et al., 2005; Teo, 2009; Teo and van Schaik, 2012; Moftakhari, 2013). On a similar note, Scherer et al. (2019) held that an individual is more likely to use technology if he/she has higher technological self-efficacy. To be specific, students with teacher affective supports may have stronger attitudinal beliefs and technological self-efficacy. Moreover, they may better cope with their self-directed language learning outside the classroom. Besides, in this study, the results indicated that teacher behavioral supports indirectly influenced students’ self-directed language learning through the mediating role of technological self-efficacy. This finding is consistent with Ertmer (2005) in that teacher behavioral supports could give students opportunities to observe the teacher and other peers on how to use technology to assist language learning. Lai (2015) pointed out that teacher behavior supports could give students ideas of possible useful technological resources in self-directed language learning. By absorbing and learning teachers’ or peers’ technological experiences, it is possible for students to enhance their confidence and technological self-efficacy which may exert advantageous influence on learning language independently beyond the classroom. Thus, it might be useful if, in the teaching process, teachers offer affective or behavioral supports to help build up students’ technological self-efficacy.

### Implications and Limitations

The present study theoretically established a solid foundation for the compensatory model of teacher supports toward self-directed language learning based on the university students’ sample of primarily highly engaged language learners and covered the internal factors of their technology acceptance and technological self-efficacy. From a practical point of view, the implications of this study can be depicted as follows: (1) enhancing a deeper understanding of students’ utilization of technology for language learning; (2) serving as a useful guidance on the development of intervention programs where teachers could optimize their potential roles in supporting students’ technology-based self-directed language learning beyond the classroom; and (3) reducing the number of obstacles posed in online learning by shifting students’ maladaptive obsessive engagement to self-determined engagement through teacher supports and the stimulation of students’ psychological factors.

Despite this study adopted rigorous procedures, there existed limitations. First of all, the results of this study were grounded on a comparatively small sample, which may give rise to a potential bias that will affect the degree to which these results are generalizable. The future study may entail involving a larger sample to include different types of student participants. Secondly, the simplex cross-sectional design being applied in this study may result in a common method bias. Hence, it is suggested that future study adopt multi-layered, multi-dimensional methods (e.g., the combination of cross-sectional design with longitudinal research) to enhance our understanding of the causality as far as possible.

## Conclusion

This study aimed to explore the contributions of teacher supports to students’ self-directed language learning and investigate whether three variables (i.e., perceived usefulness, perceived easy of use and technological self-efficacy) mediated these associations. The findings of this study indicated that teacher supports influenced students’ self-directed language learning mainly through perceived usefulness and technological self-efficacy while perceived easy of use had indirect mediating functions by directly influencing perceived usefulness. Thus, there was evidence from this study to suggest the significance of elevating teachers’ awareness of the substantial supports in establishing and enhancing students’ perceived easy of use, perceived usefulness and technological self-efficacy so that students are highly motivated to conduct self-directed language learning beyond the classroom. This study also suggested that the improvement of students’ technology-based self-directed language learning may be most feasible by promoting beneficial harmonious engagement through teacher supports and the stimulation of students’ psychological factors. Against the background of technology integration redefining teacher-student interactions in the teaching landscapes, the results of this study would inform that the future research on teachers’ compliance in relation to technology use be converted from institutional mandates into teachers’ conscientious behaviors.

## Author’s Note

XP is an associate professor in Xingzhi College, Zhejiang Normal University, Jinhua, China. His research interests are English Language teachers’ technology use for professional development and students’ learning, intercultural English education and educational psychology. His publications have appeared in International Journal of Computer-assisted Language Learning and Teaching, and Social Behavior and Personality, and Frontiers in Psychology. WC is a postgraduate student in College of Foreign Languages, Zhejiang Normal University, Jinhua, China. Her research interests are second language acquisition and technology-based self-directed learning.

## Data Availability Statement

The original contributions presented in the study are included in the article/supplementary material, further inquiries can be directed to the corresponding author/s.

## Ethics Statement

Ethical review and approval was not required for the study on human participants in accordance with the local legislation and institutional requirements. Written informed consent for participation was not required for this study in accordance with the national legislation and the institutional requirements.

## Author Contributions

XP conceived, designed, and executed the study, collected the data, wrote the manuscript, and revised the final version of the manuscript. WC analyzed the data and participated in writing and revising the manuscript. Both authors have read and approved the submitted version.

## Conflict of Interest

The authors declare that the research was conducted in the absence of any commercial or financial relationships that could be construed as a potential conflict of interest.

## Publisher’s Note

All claims expressed in this article are solely those of the authors and do not necessarily represent those of their affiliated organizations, or those of the publisher, the editors and the reviewers. Any product that may be evaluated in this article, or claim that may be made by its manufacturer, is not guaranteed or endorsed by the publisher.
